# Seasonal Variation in Mammalian Mesopredator Spatiotemporal Overlap on a Barrier Island Complex

**DOI:** 10.3390/ani14162431

**Published:** 2024-08-22

**Authors:** Timothy D. Bransford, Spencer A. Harris, Elizabeth A. Forys

**Affiliations:** 1 Animal Studies Discipline, Eckerd College, St. Petersburg, FL 33711, USA; 2 Economics Discipline, Eckerd College, St. Petersburg, FL 33711, USA; 3 Environmental Studies Discipline, Eckerd College, St. Petersburg, FL 33711, USA; 4 Biology Discipline, Eckerd College, St. Petersburg, FL 33711, USA

**Keywords:** camera trap, barrier island, *Canis latrans*, *Didelphis virginiana*, Florida, mesopredator, *Procyon lotor*, spatiotemporal overlap

## Abstract

**Simple Summary:**

In human-dominated areas where top predators no longer occur, interactions among medium-sized predators can be complex, especially when considering how they share space and time based on seasonal changes in food and habitat. We studied this using camera traps placed in various habitats from February 2021 to July 2023, at Fort De Soto County Park, a barrier island complex located in west central Florida. Three species of mammals (coyotes, raccoons, and Virginia opossums) were the most frequently photographed. Our analysis showed that during the wet season, these species were most likely to be photographed in similar habitats and times. During the dry season, when perhaps there were fewer food sources, the species shifted when they were active in a manner that created less overlap. Also in the dry season, opossums made more use of mangrove habitats. Understanding the relationships among these species is important because this area supports nesting shorebirds and sea turtles, known prey for these predators.

**Abstract:**

Due to lack of apex predators in human-dominated landscapes, mesopredator relationships are complex and spatiotemporal niche partitioning strategies can vary, especially when seasonal shifts in resource availability occur. Our objective was to understand spatiotemporal niche overlap across seasons among mesopredators inhabiting a barrier island complex. We placed 19 unbaited cameras throughout Fort De Soto County Park, Florida, USA between February 2021 and July 2023. Of six mesopredator species detected, three species had >75 detections during both the wet and dry seasons (coyote, *Canis latrans*; Virginia opossum, *Didelphis virginiana*; and raccoon, *Procyon lotor*). Using general linear mixed models, we determined that during the wet season coyote–raccoon and raccoon–opossum detections were positively associated with each other (*p* < 0.05). During the dry season, raccoon–opossum detections were positively associated, and opossums were more likely to be detected around mangroves. After calculating coefficients of overlap, we found all three species varied their temporal activity between seasons. During the dry season exclusively, all three mesopredators occupied different temporal niches. The park’s isolated but developed nature has potentially led to a destabilized mesopredator community. Understanding seasonal mesopredator dynamics of Fort De Soto is particularly important because this park supports a high number of nesting shorebirds and sea turtles, which are known food sources for mesopredators.

## 1. Introduction

Within an animal community assemblage, negative and positive ecological interactions impact both mammalian predator community structure and interactions [1,2]. Predators at higher trophic levels can regulate predators at lower levels via direct predation, exploitation competition, or by influencing behavioral changes [3,4,5]. However, these higher trophic level predators can also increase scavenging opportunities for lower-level predators, increasing resource availability [6]. In many landscapes, predators span multiple trophic levels, with a relatively small number of apex predators regulating the larger populations of mesopredators at lower trophic levels [7]. Defining “mesopredator” is complex, though, given that as apex predators decline in numbers because of anthropogenic reasons, species typically considered mesopredators rise in relative trophic level to fill this vacant niche [8,9]. Consequently, mesopredator populations can potentially experience large population growth through increased habitat and food resources, a concept named the mesopredator release hypothesis [10,11]. Additionally, behaviorally plastic species that exhibit different strategies depending on habitat and resource availability can fit both apex and mesopredator guild profiles, such as the coyote (*Canis latrans*) [12]. For simplicity, in this paper we consider species still mesopredators though they have potentially lost their apex predators and/or are considered apex predators in other locations.

Mesopredators are influenced by human activities via both top-down and bottom-up processes [13]. Direct human presence can influence the foraging patterns [14,15], home ranges [16], disturbance tolerance [17,18], and risk perception [19,20] of species, while an increase in human infrastructure modifies the landscape affecting entire ecological communities [21,22]. Human presence can also increase the availability of food resources. In fact, discarded human food waste is one of the most common dietary categories for mesopredators globally [23,24]. Thus, certain species of mammalian mesopredators, such as the coyote (*C. latrans*), red fox (*Vulpes vulpes*), raccoon (*Procyon lotor*), and Virginia opossum (*Didelphis virginiana*, hereafter opossums), have become especially adept at exploiting anthropogenic food sources [25,26].

Species occupying the same area have to partition resources in order to successfully coexist [27,28]. Competing species can generally partition their resources via three niche dimensions in order to reduce negative interactions with each other: habitat, activity times, and diet [29,30,31]. Human actions can have a strong influence on mesopredator interspecific competition; however, how a species behaviorally responds varies by context and community structure [32,33]. In areas of high recreational activity, wildlife are often pushed towards nocturnality [34,35,36], increasing the potential for niche overlap and making niche partitioning between competitors more difficult [37]. Wildlife in parks also alter their behavior to either utilize human structures when humans are least present or avoid them completely [38]. This alteration in behavior can been seen in a wide variety of ecosystems and levels of human presence [39]. With a decrease in apex predators and an increase in anthropogenic food sources, mesopredators in parks can face increased intraguild competition [40,41]. However, it is also plausible that the addition of human food creates an abundant resource that eases the burden of space and time overlap and can safely be exploited by multiple species at once [42,43].

While not as well studied, interspecific interactions can also shift with environmental seasonality across each of the aforementioned niche dimensions [32]. Depending on the type of seasonality, different niche partitioning patterns emerge. As the season shifts from wet to dry and food resources become scarcer, lions (*Panthera leo*) and spotted hyenas (*Crocuta crocuta*) experience an increase in negative interactions [44]. Subordinate species are particularly affected by seasonal changes between wet and dry periods, as dominant species respond to resource scarcity by becoming increasingly territorial and selective in their diet. This shift in behavior by dominant species often forces subordinate species to adapt their own diet and habitat use, potentially limiting their access to critical resources [45]. In areas that experience distinct summer and winter seasonality, some studies have found that mesopredator dietary niche overlap increases during the winter when the variation of food resources is limited [46]. However, other studies have found less evidence of strong mesopredator temporal niche partitioning despite observing seasonal activity shifts [47]. Seasonal presence of human activity also has the potential to influence niche partitioning, though little research overall has been conducted in this area [30,48,49].

Barrier islands are long stretches of sand and sediment that form parallel to a coastline. These islands present a unique habitat to study niche partitioning in mesopredators. Islands, when not connected to the mainland, often contain fewer species and linear food chains [7]. While many mesopredators likely have the ability to swim to barrier islands from the mainland, the frequency of these movements is often relatively low [50,51]. Instead, bridges connecting these islands to the mainland have likely increased the number of species and individuals [52,53]. During this same time, due to hunting and habitat loss, mammalian apex predators decline [54]. This results in a high density of mesopredators in an isolated environment [55].

Given that mesopredator niche overlap can be influenced by both top-down and bottom-up factors that include human disturbance, competitor presence, and seasonal change, it is unclear how a mesopredator community would niche partition on a multidimensional barrier island complex. Here, we seek to understand the spatial and temporal overlap among mesopredators inhabiting a barrier island complex that is managed primarily for recreation located on the Gulf Coast of Florida, USA at the mouth of Tampa Bay (Fort De Soto County Park, hereafter Fort De Soto). Understanding the mesopredator dynamics of Fort De Soto is particularly important because this park supports a high number of nesting shorebirds and sea turtles [56,57]. Specifically, we use a camera trap survey to determine if there is a seasonal difference in mesopredator shared space, habitat use, and nightly temporal overlap. We predict that there will be less shared space, habitat overlap, and temporal overlap between the species during the dry season, suggesting more spatiotemporal niche partitioning. Conversely, in the wet season, food sources are more abundant [58,59] and the ecosystem will be able to support increased mesopredator overlap across multiple dimensions.

## 2. Materials and Methods

### 2.1. Study Area

**Figure 1:**
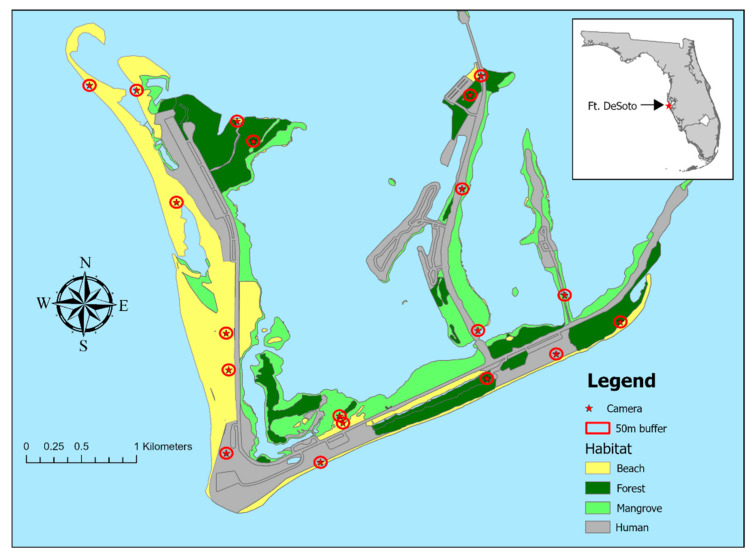
Map of habitat types across Fort De Soto County Park. Red stars represent camera trap sites and red circles represent the affiliated buffer zones.

Research was conducted from February 2021 to July 2023 at Fort De Soto, a 460 ha county-managed property located on the southern end of Pinellas County, Florida at the mouth of Tampa Bay (27.6338° N, 82.7186° W). The park consists of a barrier island and four associated keys that are linked by bridges to each other, with a singular bridge to mainland Pinellas County. The main island has a wide sandy beach with a well-established dune system, upland forested areas, and wetland areas dominated by mangroves. At the entrance to the park is a campground and throughout the park are roads, sidewalks, picnic pavilions, a historic fort, as well as some residential properties for staff (Figure 1). The park has a high visitation year-round, and averages over 2.7 million visitors, though visitation is mainly centered around Saturday and Sunday during the summer months [60]. Research on the mammalian fauna has not been published, with the exception of a study on bait-ingestion by free-ranging raccoons sampled throughout Florida [61].

Tampa Bay is located in the subtopics, which are lines of latitude between the temperate zone and the tropic zone [62]. From mid-October until mid-May, the temperature is drier and cooler. During our project, the average monthly precipitation was 24.5 mm and the average temperature was 21.3 °C. In mid-May, the tropical climate shifts north and the interaction between this warmer air and the surrounding ocean leads to the thunderstorm-dominated wet season. The average monthly precipitation was 95.8 mm and the temperature was 27.2 °C.

### 2.2. Camera Trapping

As part of a larger mammal and bird long-term monitoring project, we placed 19 unbaited solar-powered infrared-triggered cameras (Campark TC02, https://www.campark.net/ (accessed on 11 February 2024)) throughout the park on 20 February 2021 and removed them on 15 July 2023 (ca. 9400 camera trap nights). We selected camera trap sites that collectively achieved the goals of (1) having cameras throughout the park and (2) placing five cameras per habitat type (beach, mangroves, upland forest, and human-dominated). The minimum distance between two cameras was 70 m, and these cameras were in different habitat types. The average inter-camera distance was 396 m ± 12 m. The camera traps were placed on poles or trees at 0.5 m above the ground and were aimed at openings in vegetation or game trails, if possible, to increase our detection probability. We set each camera trap to operate from 2000 h to 0700 h when the park opened due to park stipulations, but this was of little concern because our mesopredators of interest were all nocturnal, and other studies have found these species will also avoid being active during the day in areas with similarly high levels of activity [15,63]. When a camera was triggered, the camera collected one 60-megapixel photograph and 15 s of 4K HD video. Our cameras had a 0.1 s trigger speed and 120° detection range, and settings used included high sensitivity and a 30 s quiet period between detections.

To enable us to look at a seasonal component for species detections, we made sure cameras were active throughout the year. Due to the dates we deployed and removed our cameras, our first dry season and last wet season were truncated. The first dry season was from 20 February to 14 May 2022, which was 11 weeks long. Wet season one was from 15 May to 15 October 2022, but all cameras had to be removed from 25 September 2022 to 8 October 2022 due to the potential threat from Hurricane Ian, so this season was 21 weeks long. Dry season two was from 16 October 2022 to 13 May 2023 (29 weeks), and wet season two was from 14 May until 15 July 2023 and was just 8 weeks long.

### 2.3. Data Organization

We collected SD cards from the cameras every two weeks and tallied all detection events with mesopredators present. For each mesopredator detection, we recorded the species, date, and time stamp available on individual photos. To avoid pseudoreplication, we followed standard protocol for mesopredators and considered detection events by species independent only if they were separated by 30 min or more; see [35,43,49,64,65]. We also coded whether the detection was for the seven-month dry or five-month wet season.

### 2.4. Habitat Classification

We used ArcGIS Pro 3.1.0 and the Florida Cooperative Land Cover (FCLC), Version 3.3 10 m resolution raster [66] for all spatial analysis. The FCLC is organized by habitat types in a hierarchical manner. We grouped land cover types into four classes using the second level of classification. (1) Beach habitat included the open sandy beach as well as the beach dune, and dominant dune vegetation included sea oats (*Uniola paniculata*) and sea grape shrubs (*Coccoloba uvifera*). (2) Mangroves included the densely vegetated mangrove swamp and the more sparsely vegetated scrub mangrove. In both habitats, red mangroves (*Rhizophora mangle*) and black mangroves (*Avicennia germinans*) were common. (3) Upland forests included hardwood forests dominated by mesic hammocks, upland coniferous, and pine flatwoods. Live oak (*Quercus virginiana*), cabbage palm (*Sabal palmetto*), and slash pine (*Pinus elliottii*) were the most common tree species. Finally, we created a human-dominated category that included all mowed grass, buildings (residential and commercial), and transportation (parking lots and roads). The raster layer was converted to vector format and overlaid on an updated 2023 base map in ArcGIS. The habitat polygons were edited to update any changes to the dynamic barrier island ecosystems that occurred between the creation of the land cover map and our research. We calculated the proportion of each land cover class within a 50 m radius of each location (Table 1). We chose a 50 m radius because it was smaller than the minimum distance between two locations and prevented oversampling of the ocean.

### 2.5. Detection Rates and Habitat Use

To quantify species space use across the park, we calculated the average weekly detection rates for each species at each camera trap location. First, we numbered the weeks in our study period and then averaged the weekly detection rate by summing the number of detection events and dividing by seven days. For each species, we then used a set of generalized linear mixed models split by season using an identity link function to estimate the relationship between species detection rates, habitat classification, and the presence of other mesopredator species, e.g., [65,67]. Our response variable was the focal species average weekly detection rate, and our predictor variables were the season, amount of each habitat type (beach, mangroves, upland forest, and human) within the 50 m buffer around the camera, and the average weekly detection rates for the other common mesopredator species. We included camera location as a random effect. All variables were natural log-transformed and checked for normality by visually checking residuals. We determined significance using z-values and the corresponding *p*-values (*α* = 0.05). All detection rate analyses were conducted using the package glmmTMB in Program R 4.3.2 [68,69].

### 2.6. Activity Overlap

To investigate the temporal activity overlap of species at the park, we calculated the coefficient of overlap (Δ) for: (1) each species between wet and dry seasons, and (2) overlap between pairs of species during each of the wet and dry seasons. First, we aggregated independent species detection events by season, controlled for daylight savings, and converted the time stamps to radial time. Then, we used the R package overlap to generate a probability density distribution using kernel density estimation [70,71]. We then compared distributions and calculated the coefficient of overlap, which is defined by the shared area under the curve using the smaller of the two distributions. Coefficient values range from 0, meaning no temporal overlap, to 1, meaning total overlap. Ridout and Linkie [68] recommend using one of three estimators depending on sample size. We used Dhat 4 for all analyses because our independent detection events were >75 for each species when sorted by season. As the coefficient of overlap is a purely descriptive statistic, we estimated the 95% confidence interval of the coefficient of overlap using 1000 bootstrapped samples from the distributions of each comparison. Then, based on the recommendation of Landler et al. [72], we calculated Watson’s U2 [73] to measure similarity between activity distributions [74] using the R package CircStats [75]. The Watson U2 test determined whether the circular distribution in activity between each pair of species was statistically similar.

## 3. Results

**Figure 2:**
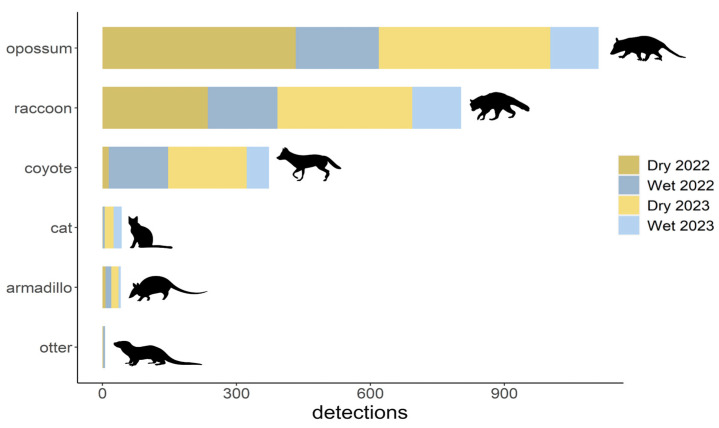
The number of independent detections by mesopredator species and season at Fort De Soto.

The 19 cameras recorded 2480 independent detections of mesopredators (Figure 2). More photos were taken during the dry season (1657 photos) than during the wet season (823 photos). As expected based on the number of weeks the cameras were active, the first dry season and the final wet season had fewer detections than then seasons that were full-length.

We observed a total of six mesopredator species over the study period, but three species were detected far more than others (Table 2, Figure 2): opossums (n_dry_ = 817, n_wet_ = 294), raccoon (n_dry_ = 538, n_wet_ = 265), and coyote (n_dry_ = 190, n_wet_ = 183). Domestic cats (*Felis sylvestris* f. catus, n_dry_ = 21, n_wet_ = 22), nine-banded armadillos (*Dasypus novemcinctus*, n_dry_ = 23, n_wet_ = 18), and river otters (*Lontra canadensis*, n_dry_ = 2, n_wet_ = 4) were relatively rare. Given that we focused on species with >75 detections per season, we have restricted our analyses to the opossum, raccoon, and coyote. We were unable to identify individuals for all observations, but given that we were able to distinguish differences between many individuals through markings, body size, and specific characteristics like leg limps across all our sites, we are confident our analyses are biologically relevant.

### 3.1. Seasonal Influences on Species Presence and Habitat Usage

**Figure 3:**
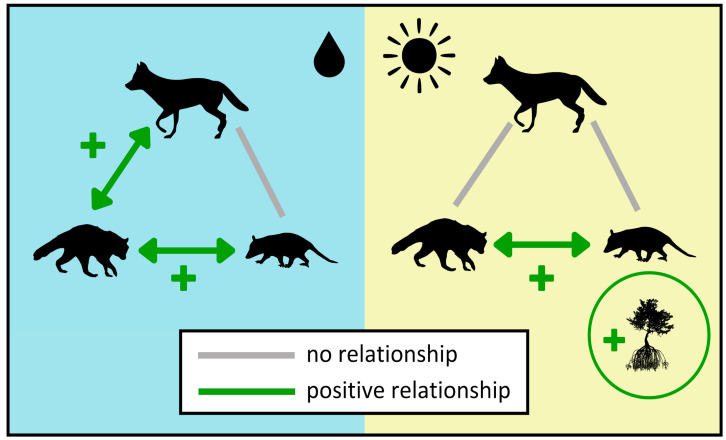
Conceptual relationships from the GLMM outputs, split by season. Wet season is on the left and the dry season is on the right. Green lines represent a positive relationship.

The generalized linear mixed model (GLMM) predicted the number of detections of these species based on habitat and the presence of other mesopredator species (Table 3; Figure 3). No predictor variables were found to be significant in the coyote dry season GLMM, but coyote detection rate had a positive significant relationship with raccoon detection rate in the wet season GLMM (*p* < 0.05). This same relationship was also positively significant in the raccoon wet season GLMM (*p* < 0.05). In all raccoon and opossum GLMMs, raccoon detection rate and opossum detection rate had a positive significant relationship (*p* < 0.01). During the dry season, opossums were more likely to be detected in locations with mangroves (*p* < 0.05).

### 3.2. Seasonal Influence on Activity Patterns and Temporal Overlap

**Figure 4:**
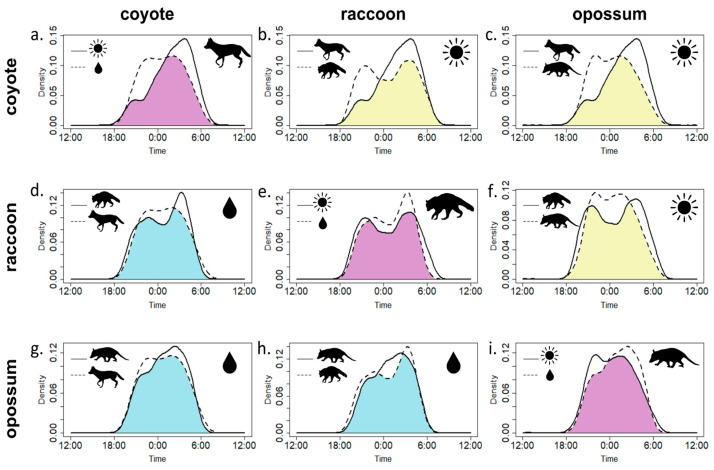
Overlap between dry and wet seasons (**a**,**e**,**i**) and between pairs of species in the dry season (**b**,**c**,**f**) and wet season (**d**,**g**,**h**). The purple shaded area indicates areas of overlap between seasons, the yellow shaded area shows overlap during the dry season, and the blue is during the wet season.

We found that all three species varied their activity patterns between seasons (Table 4; Figure 4a,e,i), and when we compared activity overlap between species, we found significant temporal niche partitioning across species during the dry season but not during the wet season (Table 4; Figure 4b,c,f). During the dry season, coyotes had a singular activity peak in the morning around 0500, while both raccoons and opossums had two peaks. Raccoons maintained a crepuscular activity pattern with fairly even peaks in the late evening around 2100 and again in the morning around 0500. Opossums showed a more nocturnal activity pattern overall but had less pronounced peaks around 2200 and 0200. During the wet season all three species had increased temporal niche overlap (Table 4; Figure 4d,g,h). Coyotes were more active throughout the night, showing more of a plateau in activity centered on 0000. Raccoons again showed a crepuscular activity pattern but had a much more prominent morning peak around 0400. Opossums also maintained their nocturnal activity pattern, with a peak in activity around 0300.

## 4. Discussion

Our results indicate that mesopredators at Fort De Soto are niche partitioning across some but not all dimensions, and there is a seasonal influence. Florida does not experience the temperature shifts more northern areas face, nor does it regularly have snow cover, so our study provides a unique look into behavioral variation across a year. The three species sampled seemingly partition their active periods during the dry season but not the wet season. We did not observe niche partitioning in terms of habitat use. This lack of significance for most habitat variables as predictors in each GLMM suggests that each species is utilizing all available habitats regardless of season and indicates that species are likely more influenced by resource presence than a habitat-specific characteristic. Across temperate and tropical environments, seasonal resource availability has been shown to influence mesopredator behavior and space use [76,77]. This idea is also supported in our study by the positive relationships with other species’ detection rates, which suggests a specific dyad of species uses the same physical space, regardless of habitat type. Given that there are no negative relationships between species detection rates, our data do not indicate there is any significant direct predation or explicit spatial avoidance between mesopredators. Put together, these results mostly support our prediction that mesopredators during the dry season have less overlap and are more likely to niche partition.

Much of the observed niche partitioning may be due to dietary overlap as all three species are opportunistic omnivores, although they are from different phylogenies and vary in size [78,79]. Previous studies show that phylogenetic relationships between species are not a primary force in shaping mesopredator interactions [76,80], and prey do not vary response by predator phylogenies [81]. Coyotes are the largest of the three species ranging from 12–15 kg in the southeastern United States [82]. Raccoons are smaller, weighing 7–9 kg [83] and the opossum is the smallest species, weighing 2–4 kg [84]. Despite the differences in these species sizes, research has also shown the body size may not be a strong driving force in mammalian mesopredator relationships [85]. All of these species are small enough to be able to meet their energetic demands with an omnivorous diet of small vertebrate prey, invertebrate prey, and plant material, though coyotes are close to the 15–20 kg threshold where the energy needed to catch small prey might exceed the caloric return [86].

Coyote diet in Florida varies by age, sex, body mass, season, and location [87]. In a study conducted throughout the state, opossums were the most commonly occurring item in coyote gastrointestinal tracts, followed by vegetation, feral hogs (*Sus scrofa* f. domestica), raccoons, and many other species [87]. In a study carried out at a more inland site < 65 km from Fort De Soto, coyote diet varied between a more natural area and a suburban area, and by wet and dry season [88]. However, there was little evidence the coyotes consumed raccoons or opossums, instead primarily foraging on vegetative matter, insects, and rabbits. Coyotes in southern latitudes of the United States are known to consume berries and other vegetative matter preferentially when available [59], and when in more suburban areas commonly consumed anthropogenic waste [88]. This pattern of consuming smaller, easier to handle prey may be related to the positive relationship between coyote size and latitude [89]. Larger coyotes tend to consume larger prey and more mammals than smaller coyotes [90]. Raccoons are omnivores that vary their diet depending on both their habitat and season [91]. Their diet in coastal ecosystems usually contains crustaceans, mollusks, fish, turtles, birds, mammals, and plants. Similar to coyotes, raccoons in more suburban areas are more likely to eat plant material and human refuse [92]. Opossums tend to eat smaller prey than coyotes and raccoons, such as invertebrates and plants, but will eat birds and mammals [93]. A comparison of opossum diet in an area with raccoons and another area where raccoons were removed found no significant differences in the proportion of food items taken, but recorded an increase in the number of opossums and an expansion of their habitat in the area without the raccoons, suggesting dietary competition [93,94]. Given that we saw no evidence of direct predation amongst any of our three focal species, we can presume that the species share at least some dietary overlap at the park throughout the year, with particularly strong overlap between coyotes—raccoons and raccoons—opossums. Ripe fruit is highest in the wet season in Florida, a food type shared between our three focal species and many of their prey species [58].

### 4.1. Seasonality and Temporal Partitioning

Our results show the primary niche partitioning mechanism between the three species is by shifting their nocturnal activity. Our study yielded high overall overlap coefficients (>0.70) for the collective data. However, it is important to understand that our sampling was limited to the period from dusk to dawn (11–12 h). If we had sampled across the full 24 h, the differences we observed would likely be more pronounced. There is much recent interest in understanding mesopredator temporal niche dynamics in human-dominated areas, especially understanding the relationship between two of the most common mesopredators, coyotes and raccoons (see Sévêque et al. 2022 [33] for a review of recent literature), and we find a strong seasonal influence as each species shifts their activity. While most studies do not take into account wet–dry seasonal differences, those that do have less clear patterns. For instance, in the Masoala-Makira landscape of Madagascar, only certain mesopredators shift their temporal activity across wet and dry seasons and this varies by study site [80]. The relationships between African carnivores on a tropical savannah are dynamic across seasons and respond to movement patterns, resource availability, and threat level [45]. Our results are much clearer and show that mesopredators as a group in the park have significantly different active periods during the dry season but not the wet season.

The dry season appears to be when the greatest pressures are exerted on our focal species, likely due to a decrease in food availability, and this is why we see coyotes shifting to the early morning hours while both raccoons and opossums shift their activity more towards the early night. Raccoons have fairly uniform crepuscular peaks, while opossums are nocturnal but decrease activity as dawn approaches. These patterns generally fit what has been observed with other populations of raccoons and opossums [95]. As mentioned earlier, natural food items are less readily available in the dry season compared to the wet season. Marsh rabbits (*Sylvilagus palustris*) are a common prey species of coyotes and other mesopredators in Florida [88] and are routinely active during dawn hours but not dusk. Additionally, they are more active during the dry season while searching for mates and reproducing [96]. The presence of marsh rabbits could shift coyote behavior to the pre-dawn hours, which in turn causes a shift in raccoon and opossum behavior to post-dusk hours. Collectively, this suggests that even though there is no direct predation, coyotes are still dominant to other mesopredators in the park.

We did not observe any significant differences between species in the wet season, but several year-round patterns did emerge. Coyotes and opossums exhibit a strong peak in nocturnal activity, remaining active throughout the night. In contrast, raccoons are primarily crepuscular, with a preference for the early morning hours. Our wet season results tend to agree with other studies looking at mesopredator overlap. Coyotes and raccoons in several areas show increased temporal overlap and habitat use as human use increases [30,35,38,97]. As previously suggested [30], it is possible that raccoons are not fearful of coyotes in high-use areas because humans exist as a more fear-inducing predator. We did not find any evidence of raccoons being fearful of coyotes, and while human presence at the park is limited to the daytime, it is still possible that the large attendance numbers for the park result in fear of humans. Coyotes and opossums also show more temporal overlap in more anthropogenic landscapes [35], but there is less agreement on temporal niche partitioning with raccoons and opossums. Previous studies show both temporal niche partitioning exists [95] and does not exist [98] between opossums and raccoons, and that the two species show multiple responses to levels of human development [35,63]. Our wet season results indicate that while raccoons are more crepuscular, they do overlap substantially with opossum active periods, presumably to exploit the same food resources. Interestingly, despite no significant differences during the wet season, we saw coyote activity throughout the night, starting after dusk, while we saw more raccoon and opossum activity in the early morning hours. This fits the idea that coyotes are the dominant mesopredator in the park, and future research should further explore competition between the three species in the wet season.

### 4.2. Species Space Use and Seasonality

We found two patterns spanning across seasons in species detection rates that help understand shared space use amongst mesopredators. First, regardless of season, raccoons and opossums were more often detected in areas where the other existed. This is not surprising as raccoons and opossums are known to indirectly compete for food and other resources in the same microhabitats [94]. Second, we found that coyote and opossum detection rates were not related to each other, regardless of season. Both species can thrive in urban and suburban landscapes, supported by anthropogenic resources like food waste [8,84,99]. Fort De Soto, while human-dominated in activity, does not resemble urban or suburban development. Coyotes and opossums succeed in urban landscapes using different strategies, and each needs a different set of anthropogenic resources [100]. Opossums succeed in urban environments because of the shelter spaces provided by buildings, while coyotes are seemingly regulated by their ability to move through the landscape [43]. The opportunities for opossums are therefore different in the park, as it both has a lower density of human structures overall and is only connected to other human development by a lone bridge. Coyotes, on the other hand, can thrive given the park provides both food resources and space to range.

We only found one species detection rate pattern that varied by season: coyotes and raccoons were more often detected in the same area during the wet season but not the dry season. This is an intriguing result since other work has shown that coyote–raccoon home range overlap does not vary by season [85]. Both coyotes and raccoons are highly adaptable generalist species that can survive in multiple habitats, developing strategies that best fit the context available to them [101,102]. Many consider raccoons to be subordinate predators to coyotes, or even prey species, but the nature of the relationship between coyotes and raccoons appears to be nuanced. In the western United States, raccoon detection rates decrease when coyotes are present in an area, suggesting predation by coyotes [43]. However, in the eastern U.S., raccoons do not seem to be fearful of coyotes and have low levels of vigilance in coyote-inhabited areas [97]. Given our lack of evidence for predation, it is likely that coyotes and raccoons are sharing food resources during the wet season when food is more plentiful.

We recorded twice as many detection events in the dry season as we did in the wet season, a pattern generally held across all our focal species. Considering the dry season is only two months longer than the wet season (seven months vs. five months), this dramatic increase in detections across the park could signify that coyotes and other species are ranging further during the dry season, as seen in opossums [103] and resident coyote populations in the southeastern United States [104]. Coyote breeding behavior is one possible explanation for both the seasonal difference in coyote detections and seasonal relationship with raccoon detection. Coyotes are seasonal breeders, mating during the winter months and giving birth in late spring–early summer [105]. They tend to reduce their daily travel during pup-rearing in the summer, which coincides with Fort De Soto’s wet season. While coyotes seasonally breed, raccoons in Florida are able to mate year-round, which is different from most other parts of their range where breeding is much more seasonal [106,107]. This difference in breeding phenology could help explain the seasonal shift in detection patterns and space use between coyotes and raccoons.

### 4.3. Seasonality in Habitat Associations

The only species–habitat type association we found was a positive relationship between opossums and mangroves during the dry season. This is understandable given the park does not have large swaths of specific habitats, making it difficult to sample habitats more in-depth, and the park shape makes it difficult for animals to avoid roadways, something many mammal species prefer [100]. Opossums, which have the smallest home range of our three focal species [108], are known to be generalist omnivores that exist in a multitude of habitats [22,109]. There are several previously recorded instances of opossums taking advantage of mangrove habitats [110,111]. Mangroves at Fort De Soto routinely have dead fish and other animals that are available for scavenging. During the dry season, opossums might lose out on scramble competition with raccoons for food acquisition given they forage at similar times [98] and in turn fall back on scavenging in mangrove habitats when needed to avoid more drastic behavioral changes. Interestingly, though, in the detection events where we observed multiple species simultaneously, opossums were most often the mesopredator winning a direct antagonistic interaction with both raccoons and coyotes. Coyotes are known to predate on opossums, but this varies again by location [12,112] and, as human development increases predation on opossums by coyotes, is predicted to decrease [22]. Combined with our temporal partitioning results, it seems that opossums are a subordinate species to raccoons and coyotes and slightly shift their niche during the dry season when food competition is higher.

### 4.4. Considering Other Mesopredators

We did regularly detect other mesopredators at sample sizes that were too small to be included in our models, and they undoubtedly have some degree of influence on the park’s mesopredator community. Domestic cats were detected 43 times, primarily at our most human-dominated location, the historic fort, and nine-banded armadillos were detected 41 times, primarily in the dunes, marshes, and forests. Both species are not native to Florida. Domestic cats at Fort De Soto are most likely unwanted pets abandoned at the park and are potential competitors with all three of our modeled species and might be prey species for coyotes [113]. Research has shown cats can significantly decrease the biodiversity of natural areas, particularly islands [114]. The nine-banded armadillo’s range has expanded over the last several decades through both natural extension and translocation by humans and is now throughout the southeastern United States [115]. Competition between armadillos and our three modeled species is possible at the smallest end of the prey-size continuum. Armadillos primarily eat arthropods but will also take small vertebrates and carrion [116].

There are several other non-mammalian vertebrates that may also compete with our three model organisms, particularly for small and medium-sized prey. Eastern diamondback rattlesnakes (*Crotalus adamanteus*) are frequently seen at Fort De Soto but would probably not be detected by our infrared camera traps because they are ectothermic and their body temperature often matches the background surface temperature [117]. In a study conducted in Florida, rattlesnakes primarily consumed rabbits and other small mammals [118]. Great-horned owls (*Bubo virginianus*) nest throughout the park and were detected several times by our cameras when they came to the ground to get prey. Owls frequently also eat small and medium mammals in addition to birds [119].

What is also missing from the mammalian predator community at Fort De Soto are apex predators like the red wolf (*Canis rufus*) and the Florida panther (*Puma concolor*) and mesopredators such as bobcats (*Lynx rufus*), gray foxes (*Urocyon cinereoargenteus*), red foxes (*Vulpes vulpes*), and striped skunks (*Mephitis mephitis*) that are rarely found in Pinellas County [120]. Pinellas is the most densely populated county in Florida, with approximately 94.7% of the county classified as urban or suburban, 0.3% as rural, and 5% set aside for parks and preserves [121]. The county is a peninsula and Fort De Soto occurs at the southernmost point, so these non-volant mammals would need to travel from more natural areas north of the county through this highly urbanized county to reach Fort De Soto. While mesopredators in urban areas might have strategies and sufficient resources to abate the need for intense competition [63], we can still see a shift in mesopredator community assemblage due to species-specific characteristics. The urban buffer surrounding the park and the modified nature of Fort De Soto’s environment, with a combination of natural and anthropogenic features, has potentially led to a destabilized mesopredator community. Species more tolerant to humans are able to thrive on the high levels of anthropogenic resources provided while less tolerant species cannot survive, as suggested by Sévêque and colleagues [32]. Without the immigration of other mesopredator species, coyotes, raccoons, and opossums have firmly established themselves in the park.

### 4.5. Management Implications

Understanding the relationship among mesopredators inhabiting barrier islands connected to mainlands is of particular importance because these species are a significant threat to nesting seabirds, shorebirds, and sea turtles [52,122,123,124,125]. Most coastal and marine birds that nest on open, sandy beaches have declined during the past century due to habitat loss, disturbance, global environmental change, and an increase in non-native or more abundant native mesopredators that predate on eggs and chicks [126,127]. Every species of sea turtle is now federally listed in the United States due a similar list of threats, and predation by mesopredators of eggs and even hatchlings can significantly lower productivity [128,129].

The 4.6 km of open beach at Fort De Soto provides critical nesting habitat for shorebirds and seabirds such as American oystercatcher (*Haematopus palliates*), black skimmer (*Rynchops niger*), least tern (*Sternula antillarum*), snowy plover (*Anarhynchus nivosus*), and Wilson’s plover (*Charadrius wilsonia*). The majority of these species are listed as Threatened by the State of Florida [130]. Wilson’s plovers are not yet listed by the state but have been designated as a bird of conservation concern by the U.S. Fish and Wildlife Service [131]. In the past decade, the majority of egg and nest predation at Fort De Soto has been attributed to mesopredators, primarily coyotes, followed by raccoons and one documented instance of an opossum [132]. Loggerhead sea turtles (*Caretta caretta*) nest at Fort De Soto, and this species is listed as threatened under the United States Endangered Species Act [133]. While site-specific data are not available, coyotes and raccoons are the two dominant predators of loggerhead sea turtles elsewhere in Florida [124,128,129].

Non-lethal measures such as placing screens or cages around loggerhead and plover nests have successfully increased egg survival, but incidences of nest abandonment by plovers have occurred [129,134] and these methods do not protect hatchlings and chicks [135]. Predator control can be a successful tool, but a thorough understanding of mesopredator dynamics is needed. Our current research did not find any negative relationships between mesopredators, which may indicate that controlling one predator is not likely to cause an increase in others. Particularly during the wet season when the birds and turtles are nesting, there appears to be little spatial or temporal separation among our mesopredators perhaps because resources are abundant.

## 5. Conclusions

Our study provides evidence that mammalian mesopredators shift their spatiotemporal niche partitioning by season. Fort De Soto presents a unique opportunity to study mesopredator niche partitioning because it has seasonal food availability but is isolated from other natural habitats and provides high levels of anthropogenic resources year-round. This has led to a destabilized mesopredator community that is comprised of large populations of three human-tolerant species: coyotes, raccoons, and opossums. Coyotes seem to be the dominant mesopredator and influence the behavior of other species to varying degrees. Most niche partitioning is temporal and manifests during the dry season. Our study has several limitations that can easily be addressed in future research. First, we were prevented from sampling during the day due to park regulations. While it is highly unlikely that our focal species are significantly active throughout the day, future studies should strive to sample for a 24 h period. Second, our study only focused on spatial and temporal niche partitioning, but species often also partition through food selection. Given the significance of season, future research should focus on food partitioning in similar systems to better understand the driving factors of spatiotemporal niche partitioning. Our study had fairly stringent criteria for determining which species to include, and future work could expand to identify specific individuals and include the influence of other mesopredators as well, including ones not as easily detected like rattlesnakes. This could be possible by increasing the number of camera traps per habitat type. Our study relied on relatively few cameras per habitat type due to project constraints, and increasing the number of cameras overall would potentially increase detection rates for species that use a habitat in a more subtle way than we anticipated. Given this, we tried to limit the scope of our conclusions to fit with our study design. Mesopredators play a crucial role in habitats affected by human actions and, given their behavioral plasticity, can greatly impact the ecological relationships found within an ecosystem. Large mesopredator populations supported by anthropogenic resources have the potential to severely hamper conservation initiatives of threatened species living in the area, especially on barrier islands with limited access. Incorporating the influence of seasonality into similar projects will provide better information for researchers, conservationists, and park managers alike.

## Figures and Tables

**Table 1:** The amount of each habitat class in the 50 m buffer around the camera location, given as a percentage of the total area.

Location	Beach	Mangrove	Forest	Human	Ocean
1	63	0	0	0	37
2	62	0	0	0	38
3	34	19	37	10	0
4	0	8	92	0	0
5	100	0	0	0	0
6	100	0	0	0	0
7	100	0	0	0	0
8	0	0	0	100	0
9	27	17	0	56	0
10	0	49	3	47	0
11	0	6	0	94	0
12	0	0	99	1	0
13	1	0	0	99	0
14	20	61	0	18	1
15	100	0	0	0	0
16	0	59	0	41	0
17	14	9	0	77	0
18	0	25	0	75	0
19	9	0	100	0	0
20	0	0	20	78	2

**Table 2:** Average weekly detection rates for our focal species by season.

	Locations Observed	Dry	Wet
**coyote**	18	0.11 ± 0.01	0.22 ± 0.02
**raccoon**	19	1.26 ± 0.07	0.55 ± 0.03
**opossum**	13	0.59 ± 0.06	0.31 ± 0.03

**Table 3:** Season-specific GLMM results for coyotes, raccoons, and opossums. Significant differences are bolded.

		Wet				Dry			
Species	Fixed Effect	Estimate	SE	Z-Value	*p*-Value	Estimate	SE	Z-Value	*p*-Value
coyote	intercept	0.24	0.17	1.42	0.155	0.16	0.07	2.21	**0.027**
	beach	−0.02	0.02	−1.19	0.236	−0.01	0.01	−1.23	0.219
	mangrove	−0.02	0.02	−1.11	0.268	−0.01	0.01	−0.94	0.348
	forest	0	0.02	−0.03	0.977	0	0.01	0.17	0.863
	human	0.01	0.02	0.32	0.746	0	0.01	−0.46	0.646
	raccoon rate	0.19	0.08	2.44	**0.015**	0.01	0.05	0.28	0.777
	opossum rate	−0.08	0.14	−0.59	0.552	0.01	0.07	0.17	0.866
raccoon	intercept	−0.17	0.2	−0.85	0.397	−0.05	0.29	−0.16	0.869
	beach	0.03	0.02	1.34	0.181	0.04	0.03	1.49	0.135
	mangrove	−0.01	0.02	−0.37	0.715	−0.01	0.03	−0.21	0.831
	forest	0.03	0.02	1.8	0.071	0.03	0.03	1.07	0.284
	human	0.02	0.02	0.92	0.358	0.03	0.03	0.95	0.342
	coyote rate	0.67	0.25	2.69	**0.007**	0.19	0.66	0.28	0.777
	opossum rate	0.56	0.2	2.84	**0.005**	0.81	0.21	3.84	**<0.001**
opossum	intercept	0.12	0.21	0.59	0.552	0.03	0.24	0.13	0.898
	beach	0	0.02	−0.13	0.899	0.00	0.02	−0.2	0.845
	mangrove	0.02	0.02	1.2	0.232	0.05	0.02	2.28	**0.022**
	forest	0.01	0.02	0.65	0.515	0.03	0.02	1.24	0.215
	human	−0.02	0.02	−0.7	0.485	−0.03	0.02	−1.24	0.216
	coyote rate	−0.14	0.23	−0.6	0.548	0.09	0.43	0.21	0.837
	raccoon rate	0.25	0.1	2.39	**0.017**	0.33	0.1	3.24	**0.001**

**Table 4:** Results from activity overlap analyses; 95% confidence intervals are from 1000 bootstrapped samples for the designated distribution comparison. Watson’s U2 test statistics are presented with their corresponding *p*-values, and statistically significant differences are presented in bold.

Season	Species 1	Species 2	Δ	95% CI	Watson’s U2	*p*-Value
dry and wet	coyote	coyote	0.81	0.72–0.89	**0.41**	***p*** **< 0.001**
dry and wet	raccoon	raccoon	0.89	0.84–0.94	**0.36**	***p*** **< 0.01**
dry and wet	opossum	opossum	0.90	0.85–0.96	**0.24**	***p*** **< 0.05**
dry	coyote	raccoon	0.81	0.74–0.87	**0.60**	***p*** **< 0.001**
dry	coyote	opossum	0.78	0.71–0.85	**0.94**	***p*** **< 0.001**
dry	raccoon	opossum	0.85	0.80–0.89	**1.06**	***p*** **< 0.001**
wet	coyote	raccoon	0.91	0.85–0.97	0.12	*p* > 0.10
wet	coyote	opossum	0.94	0.90–1.00	0.07	*p* > 0.10
wet	raccoon	opossum	0.92	0.87–0.99	0.15	*p* > 0.10

## Data Availability

The raw data supporting the conclusions of this article will be made available by the authors on request.
